# Genome-wide association study uncovers major genetic loci associated with flowering time in response to active accumulated temperature in wild soybean population

**DOI:** 10.1186/s12864-022-08970-2

**Published:** 2022-11-11

**Authors:** Guang Yang, Wei Li, Chao Fan, Miao Liu, Jianxin Liu, Wenwei Liang, Ling Wang, Shufeng Di, Chao Fang, Haiyang Li, Guohua Ding, Yingdong Bi, Yongcai Lai

**Affiliations:** 1 Postdoctoral Scientific Research Station of Heilongjiang Academy of Agricultural Sciences, Harbin, 150028 Heilongjiang China; 2 Institute of Crop Cultivation and Tillage, Heilongjiang Academy of Agricultural Sciences, Harbin, 150028 Heilongjiang China; 3 Innovative Center of Molecular Genetics and Evolution, School of Life Sciences, Guangzhou University, Guangzhou, 510405 China

**Keywords:** Genome-wide association study, Flowering time, Active accumulated temperature, Glycine soja, Haplotypes

## Abstract

Flowering time and active accumulated temperature (AAT) are two key factors that limit the expanded production especially for soybean across different regions. Wild soybean provides an important germplasm for functional genomics study in cultivar soybean. However, the studies on genetic basis underlying flowering time in response to AAT especially in wild soybean were rarely reported. In this study, we used 294 wild soybean accessions derived from major soybean production region characterized by different AAT in Northeast of China. Based on genome-wide association study (GWAS), we identified 96 SNPs corresponded to 342 candidate genes that significantly associated with flowering time recorded in two-year experiments. Gene Ontology enrichment analysis suggests that the pathways of photosynthesis light reaction and actin filament binding were significantly enriched. We found three lead SNPs with -log_10_(*p*-value) > 32 across the two-year experiments, i.e., Chr02:9490318, Chr04:8545910 and Chr09:49553555. Linkage disequilibrium block analysis shows 28 candidate genes within the genomic region centered on the lead SNPs. Among them, expression levels of three genes (aspartic peptidase 1, serine/threonine-protein kinase and protein SCAR2-like) were significantly differed between two subgroups possessing contrasting flowering time distributed at chromosome 2, 4 and 9, respectively. There are 6, 7 and 3 haplotypes classified on the coding regions of the three genes, respectively. Collectively, accessions with late flowering time phenotype are typically derived from AAT zone 1, which is associated with the haplotypic distribution and expression levels of the three genes. This study provides an insight into a potential mechanism responsible for flowering time in response to AAT in wild soybean, which could promote the understanding of genetic basis for other major crops.

**Supplementary Information:**

The online version contains supplementary material available at 10.1186/s12864-022-08970-2.

## Introduction

Flowering time determines the adaptation of crops to a wide range of latitudes [28]. Currently, the well-studied pathways are the flowering pathways in Arabidopsis and rice. There are four main regulatory pathways in Arabidopsis: photoperiod pathway, gibberellin pathway (GA pathway), vernalization pathway and autonomous pathway [2, 8]. Among them, photoperiod regulation of flowering pathway is of great significance to plant growth and development. Soybean is a typical short-day plant and is very sensitive to photoperiod response, but the photoperiod sensitivity is different among different varieties, resulting in different growth periods of soybean varieties. Soybean flowering time is an important agronomic trait that is crucial to soybean yield, quality and adaptability. According to the research methods of classical genetics, 14 loci have been identified that are significantly related to flowering stage (e.g., [6, 7, 35]), which are *E1, E2, E3, E4, E5, E6, E7, E8, E9, E10, E11, J, Tof11, Tof12*. Although these genes controlling flowering and maturity have been identified in various cultivars soybean, the genetic basis of this broad adaptation is still unclear.

Active accumulated temperature (AAT) becomes the most important factors that determine the adaptation zone of the soybean varieties [17, 29]. Breeders and farmers used daily average temperature ≥ 10 °C AAT to describe the heat requirement and determine the adaptation zone of each cultivar in some main producing regions, such as Northeast of China, Heilongjiang province. Cultivated soybeans were domesticated from wild soybeans 5000 years ago (*Glycine soja*) [3]. A common suite of traits was convergently selected during domestication including early flowering and maturity, increased apical dominance, loss of seed shattering, and reduced seed dormancy [28, 42]. Due to bottlenecks and human selection, cultivated soybeans have much lower genetic diversity than their wild counterparts [22]. This reduced variation may have resulted in the loss of some genes or alleles important for adaptation to different environments.

Therefore, compared with cultivated soybean, wild soybean has many excellent characters. For example, wild soybean has relatively high protein content, strong stress resistance, can survive in a variety of environments, has more flowers and more pods, and has strong reproductive ability. Wild soybean might contain useful genetic resources that can be re- introduced into cultivated soybeans via breeding. Wild soybean is distributed across a broad geographical range (N24 ~ 53^o^, E97 ~ 143^o^), which includes China, Korea, Russia, and Japan, and has adapted to a variety of ecological conditions [23, 32]. Therefore, the huge gene treasure house contained in wild varieties has received more and more attention from breeders in recent years.

It was speculated that the valuable genetic resources in wild soybean can provide abundant genetic resources for cultivated soybean, and mining the key genes of yield traits in wild soybean provides a theoretical basis for broadening the genetic basis of cultivated soybean, and can effectively solve the problem of cultivation. However, the genetic basis of adaptation of wild soybean to different latitudes remains completely unknown. In recent years, genome-wide association analysis has made great progress in soybean research, mainly focusing on the study of important agronomic traits such as yield, quality, and stress resistance [4, 19, 39].

In this study, we used 294 soybean accessions derived from 4 AAT zones in Northeast of China, and investigated the flowering time in 2 years in same location. GWAS results suggest some overlapped SNPs that significantly associated with different sets of flowering time in 2020 and 2021 as well as flowering time in average in 2020–2021. Collectively, we identified three differentially expressed genes in 20 soybean accessions based on qPCR. The three genes were not yet reported previously that directly related to flowering time. Haplotypes analysis suggest that most accessions in subgroup of late flowering haplotypes belong to AAT zones with high ranking (AAT 1), which reveals that AAT shapes the flowering time phenotype and fitness of wild soybean. This study provides deep understanding of molecular mechanism of wild soybean adaption to AAT, which is helpful to guide the cultivated soybean breeding.

## Material and methods

### Plant materials and flowering determination

In this study, 294 wild soybean (*Glycine soja*) accessions were collected from five AAT zones across Heilongjiang province, Northeast of China. The ranges of active accumulative temperature for AAT1 to AAT5 zones were > 2700, 2500–2700, 2300–2500, 2100–2300 and 1900–2100 °C, respectively. These accessions were planted in Apr 30th 2020 and May 5th 2021 at Heilongjiang soybean field base (N45.51^o^. E126.51^o^). Each accession containing 20 plants was sowed, and we planted in 2 rows, 2 holes in each row, and 5 plants in each hole, with 100 cm between plants within each row and 65 cm between rows. The 100 cm distance is used to avoid intertwining between each other and ensure the seed purity during harvest, with consideration of its unlimited growth habit. The air temperature in average during May and Sep in 2020 and 2021 were 20.85 and 21.09 °C, respectively. Soybean plants grown in the field were managed according to standard local agronomic practice with the following fertilizer application guideline: 48 kg N ha^− 1^, 120 kg P_2_O_5_ ha^− 1^, and 100 kg K_2_O ha^− 1^ as the basal fertilizer. Bamboo scaffoldings were used in the late stage of soybean seedlings to ensure plants upright growth. Flowering time was scored as the number of days from emergence to opening of the first flower on any node of a plant [13].

### Genome-wide association analysis

Leaves for 294 wild soybean accessions were sampled for DNA isolation. We adopted a CTAB method to extract DNA, and at least 5 μg of DNA was used to construct a sequencing library with an Illumina TruSeq DNA Sample Prep Kit, according to the manufacturer’s instructions. Paired- end sequencing (150 bp) of each library was performed on the Illumina Hi- Seq X Ten system. The paired-end sequencing reads of 294 accessions were mapped to the Williams 82 (Wm82.a2.v1) genome with BWA 0.6.1-r104 software with default parameters [38]. SNP and indel calling were performed with GATK and SAMtools ver. 0.1.19 software. SNPs with a MAF < 1% were discarded, and indels with a maximum length of 10 bp were included. SNP annotation was carried out based on the W82 genome using snpEff ver. 3.1 software [5]. In total, 37,125 biallelic SNPs, with a minor allele frequency (MAF) ≥ 5% were used in the association mapping. The values of flowering time in 2020 and 2021 were first normalized with Quantile-Quantile (QQ) plots norm function of the R software (version 3.2.1). Then, we performed Genome-wide association study (GWAS) with consideration of kinship matrix algorithms using general linear mixed model (GLM) implemented in EMMAX software [21].

To correct the population structure and reduce false positive rates, we performed a principal component analysis (PCA) on the SNP matrix of the wild soybean population and used the first 4 principal components as covariates in the GWAS analysis as reported previously [34]. The Bonferroni correction was used to determine the significant SNPs at genomic level, while a suggestive threshold was used to determine the association at lower levels to identify the haplotype variation of candidate genes [20]. A linkage disequilibrium (LD) analysis was conducted with the Haploview software (version 4.2) [1] to investigate the relatedness degree of the candidate genes to the lead SNP. The LD blocks were generated when the upper 95% confidence bounds of *r*^2^ value exceeded 0.98 and the lower bounds exceeded 0.70 [14]. The genes identified in the LD block were selected as potential candidate genes that might control flowering time.

### Screening of candidate genes using gene expression analysis

To screen the candidate genes controlling flowering time, we selected 20 accessions with contrasting flowering time values (10 early and 10 late) from experiments performed in 2020 and 2021 at Heilongjiang soybean field base. We further conducted quantitative real-time PCR (qRT-PCR) analysis (ABI StepOnePlus, Applied Biosystems Co., Ltd., USA) for the candidate genes identified by LD-block analysis. For qRT-PCR analysis, samples were collected from the top fully expanded leaves of 60-day old plants. The total RNA was extracted using Ambion PureLink RNA mini kit according to manufacturer’s instruction. Two micrograms of total RNA were reversely transcribed to cDNA with SuperScript VILO cDNA Synthesis Kit (Invitrogen Life Technologies, USA). The qRT-PCR was performed using SYBR Green PCR Master Mix (Applied Biosystems, USA) with cDNA as a template with the following cycling parameters: 95 °C for 10 s, 55 °C for 20 s, and 72 °C for 20 s. The specific primers for qRT-PCR were designed using Primer Prime Plus 5 Software Version 3.0 (Applied Biosystems, USA). The primer sequences are shown in Table S1. Relative expression of gene against actin, a housekeeping gene, was calculated as follows: 2^−ΔΔCT^ (ΔCT = CT, gene of interest−CT, *Actin1* [27];). Six biological replicates were performed.

## Results

**Fig. 1:**
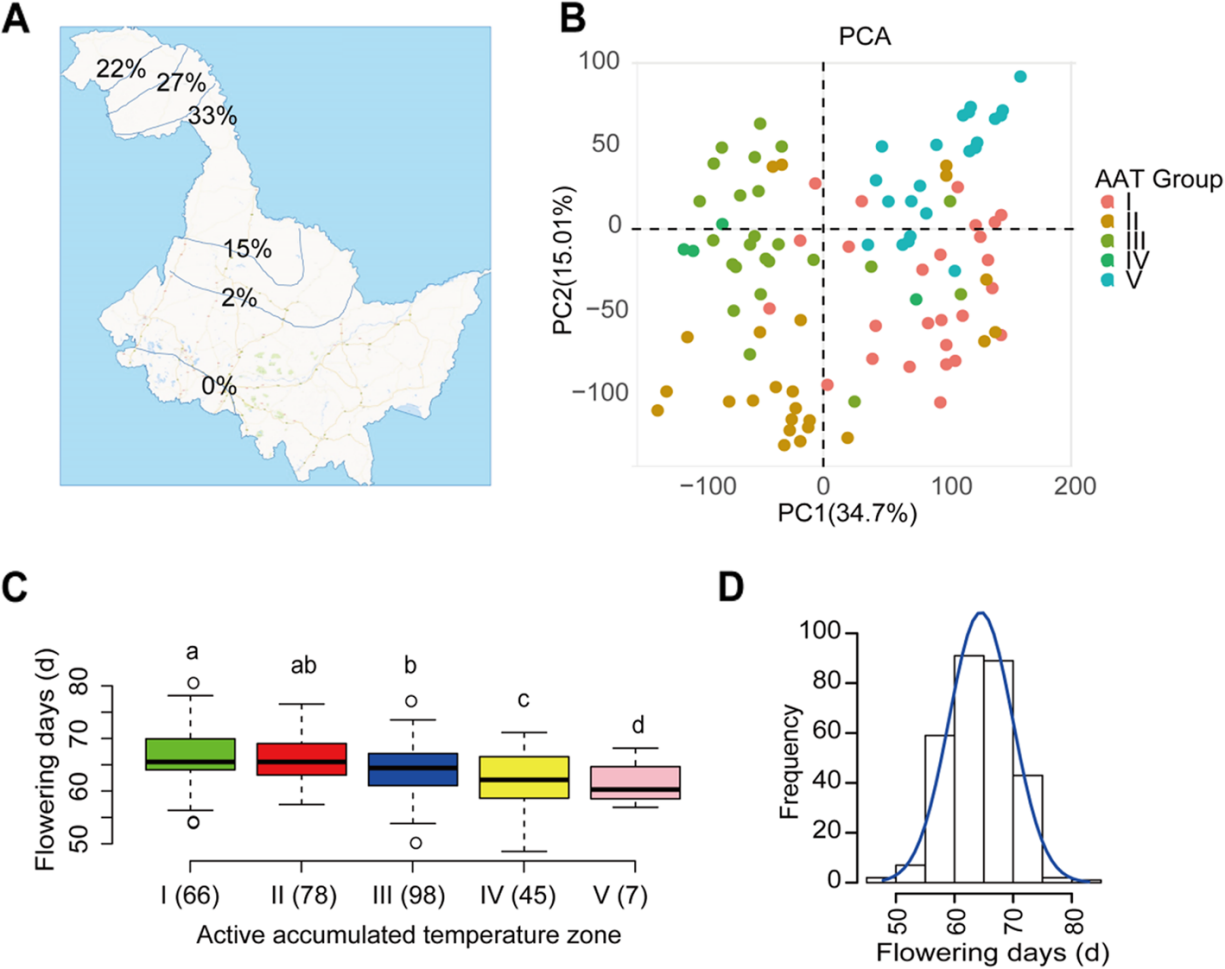
Distribution of geography and flowering days in 294 soybean accessions. **A** Map distribution of 294 wild soybean accessions collected from different active accumulated temperature zones. The numbers in the map represent the percentage of soybean accessions accounting for total accessions used in this study. Among the 294 accessions, 22, 27, 33, 15, 2 and 0% were derived from active accumulated temperature (AAT) zones from 1 to 6, respectively. **B** Principal component analysis on the 37,125 SNPs filtered in the 294 soybean accessions. **C** Distribution of flowering days among different AAT zones. The numbers of accessions in each AAT zone group were displayed in bracket. **D** Histogram of flowering days measured in 2020 in the 294 soybean accessions

In this study, 294 wild soybean accessions were collected spanning from four AAT zones of Heilongjiang province, Northeast of China (Fig. 1A; Table S2). After SNP filtering, we obtained 37,125 SNPs in this natural population as mentioned in Material and methods section. In the 294 accessions, the numbers of SNPs across chromosomes ranged from 7000 to 23,000 (Fig. S1A). Before SNP filtering, we found that the most minor allele frequencies of SNPs were ranged from 0.02 to 0.12 (Fig. S1B). The linkage disequilibrium decay (*r*^2^) across different linkage disequilibrium windows were 0.23 (Fig. S2A). Some loci were found in heterozygosity. The percentage of heterozygosity was ranging from 3 to 72%, with a mean of 33% among the 294 wild soybean accessions (Fig. S2B). Principal component analysis suggests that the PC1 and PC2 explain 34.7 and 15% phenotypic variance of the soybean population structure, respectively (Fig. 1B).

**Fig. 2:**
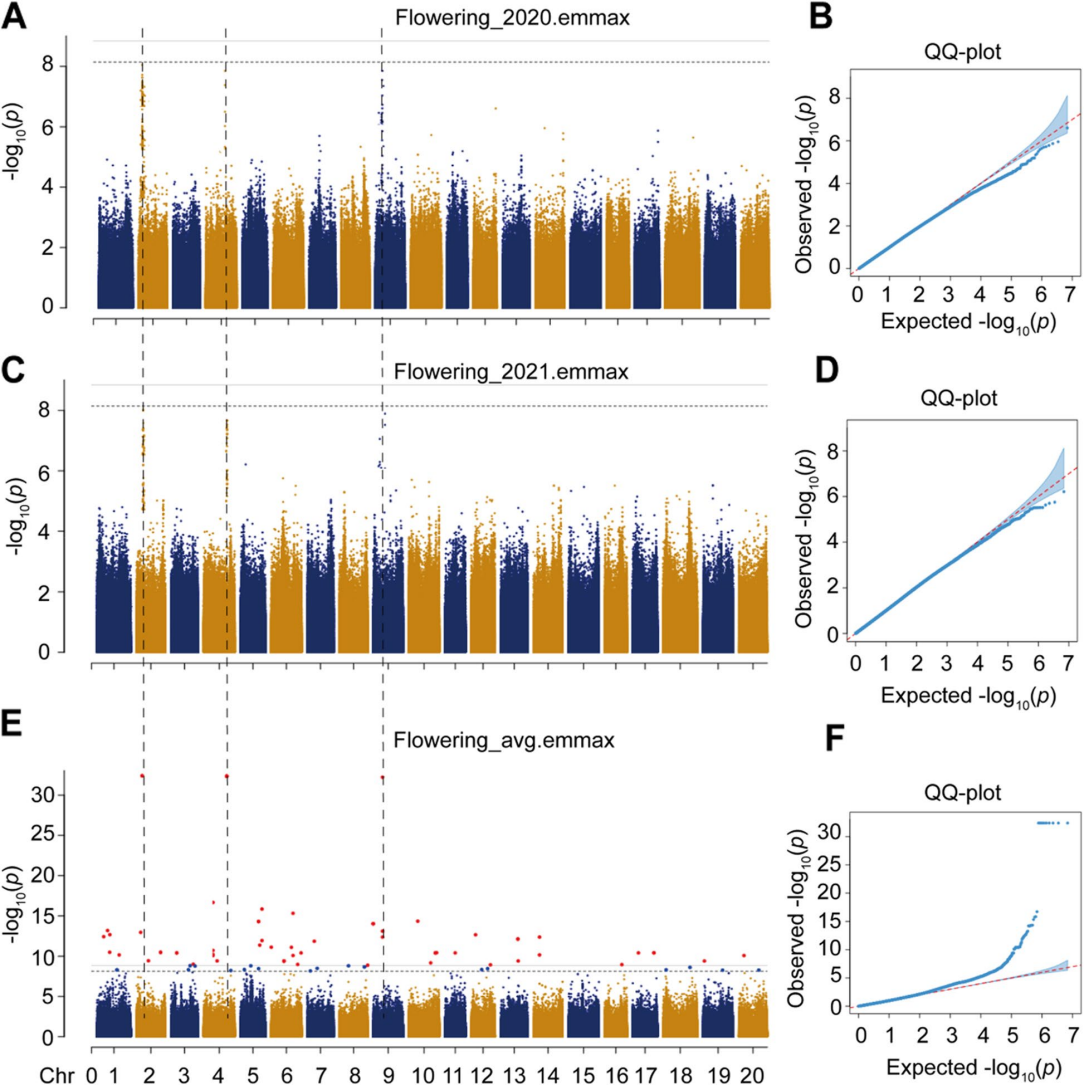
Genome-wide association study on flowering time recorded from different years. Manhattan plots of flowering days measured in 2020 (**A**), in 2021 (**C**) and in average of data in 2020 and 2022 (**E**). QQ plot of flowering days measured in 2020 (**B**), in 2021 (**D**) and in average of data in 2020 and 2022 (**F**). The dotted lines across panels **A**, **C**, **E** represent the overlapped genomic regions surrounding the SNPs that are significantly associated with each trait

Flowering time was recorded based on 2 years observations in the field in the 294 wild soybean populations. The flowering time in average across 2 years experiments generally decreased with the AAT zones (Fig. 1C). The flowering time were ranged from 50 to 80 days in the soybean population (Fig. 1D; Table S2). Additionally, the trend of flowering days decreased following AAT zones is also true for each year experiment (Fig. S3A-B). In particular, the variation of flowering time for 2020 and 2021 in the population follows a normal distribution (Shapiro-Wilk; *P* < 0.05), and the values were ranged from 50 to 75 days and ranged from 45 to 80 days, respectively (Fig. S4A-B; Table S2). The Pearson correlation coefficient (*r*) of flowering time between 2 years experiments was 0.27 (*p* < 0.05) (Fig. S5; Table S2). The heritabilities of flowering time in 2020, 2021 and their average were 0.42, 0.33 and 0.46, respectively (Table 1). To further uncover the candidate genes responsible for flowering time in 2020, we performed a genome-wide association study (GWAS). Results show that EMMAX using LRM model has strong prediction with observed values for all three sets of data including flowering time in 2020, flowering time in 2021 and flowering time in average across 2 years (Fig. 2).

**Table 1:** Variance component analysis with marker-based heritability estimates

Trait	h^2^	SE of h^2^	V_g_	V_e_
Flowering in 2020	0.4181	0.0678	17.9331	31.142
Flowering in 2021	0.3277	0.0113	856.1219	2126.298
Flowering in average	0.4633	0.0113	3279.893	4698.303

**Fig. 3:**
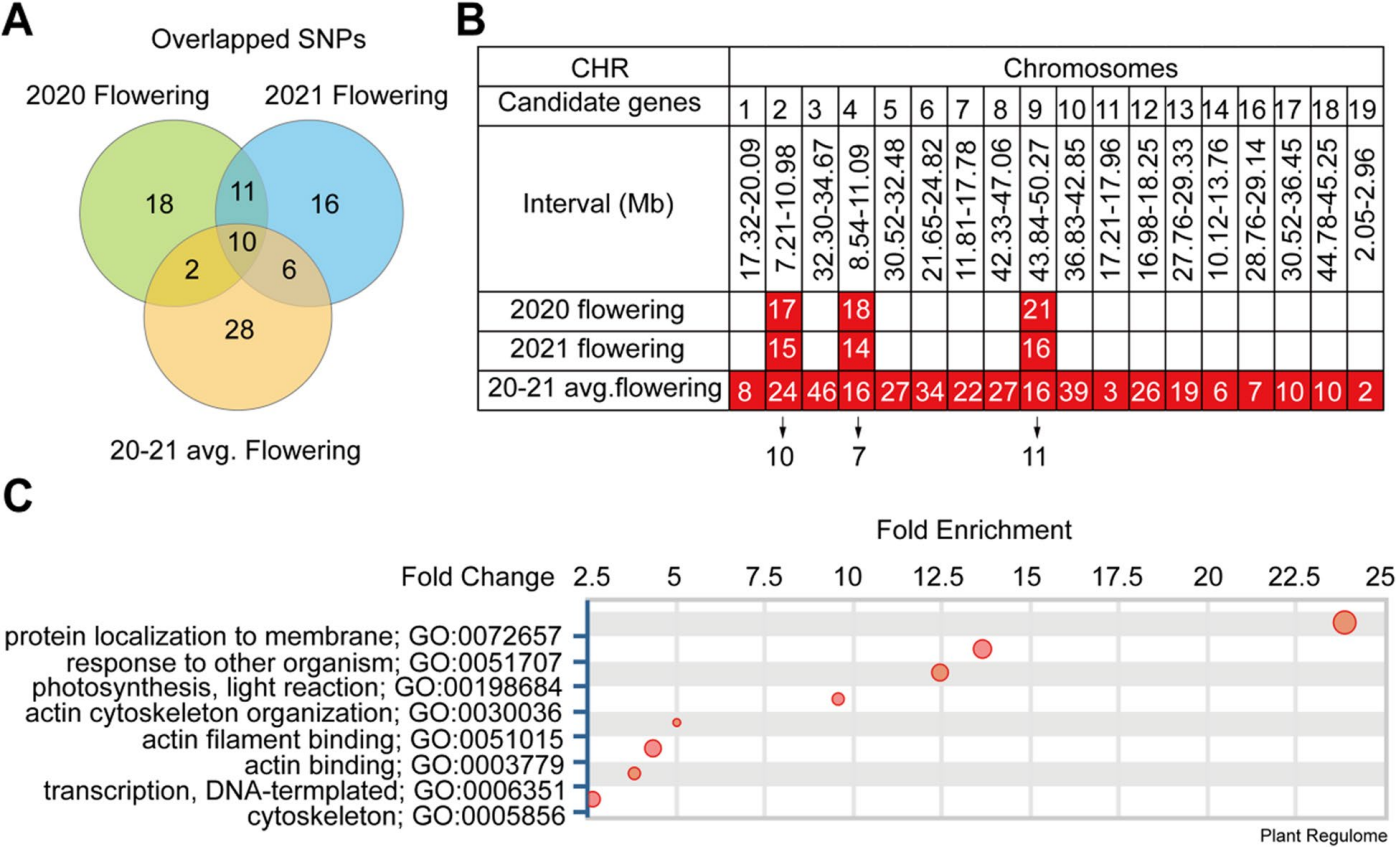
Overlapped SNPs across flowering phenotype recorded from different years in 294 soybean accessions. **A** Overlapped SNPs among the flowering time in 2020, in 2021 and flowering time in average in 2020 and 2021. **B** Table of overlapped SNPs and their corresponding genes at different chromosomes. The numbers in red cells represent the numbers of genes surrounding the overlapped SNPs. **C** Gene Ontology analysis on the 342 candidate genes corresponded to the 92 SNPs that significantly associated with the flowering time in average in 2020 and 2021

Based on suggestive threshold, we found that there are 74, 70, and 92 SNPs that significantly associated with flowering time recorded in 2020, 2021 and flowering time in average between the 2 years experiments, respectively (Fig. 3A; Table S3). In particular, we found 10 overlapped SNPs including three lead SNPs with -log_10_(*p*-value) > 32 that were corresponded to 28 candidate genes across two-year experiments (Fig. 3A-B). Totally, we identified 342 genes that corresponding to the genomic region surrounding ~ 50 kb of the 92 SNPs that significantly associated with flowering time in average between 2020 and 2021 (Table S4). Furthermore, we used these genes to perform Gene Ontology analysis, and found that serval pathways are significantly enriched (Fig. 3C), including protein localization to membrane, response to other organism, photosynthesis, light reaction, actin filament binding, acting binding, transcription, DNA-templated, and cytoskeleton (Fig. 3C). The 28 candidate genes distributed at chromosome 2, chromosome 4, and chromosome 9 were further analyzed (Fig. 3B; Table S5).

**Fig. 4:**
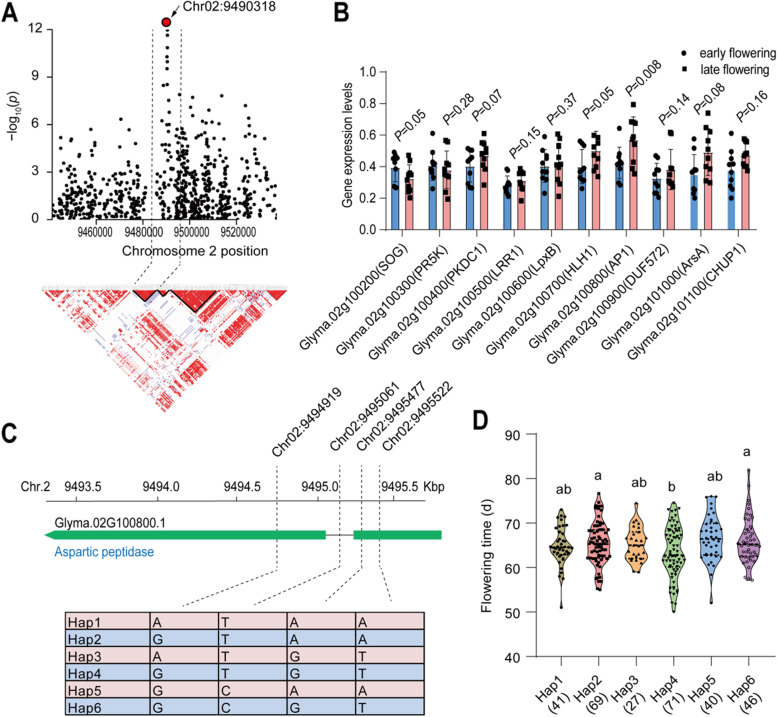
Haplotype analysis of aspartic peptidase at chromosome 2. **A** Zoom-out Manhattan plot of flowering days in average of 2 years at chromosome 2. The dotted lines represent the linkage disequibriurm (LD) (~ 32.83 kb) window surrounding the peaked SNP (Chr02:9490318). The red circle represents the lead SNP with -log_10_(*p*-value) = 33.42. **B** The relative gene expression levels of ten candidate genes within the LD window in 20 wild soybean accessions. The 20 accessions in two groups were characterized by their contrasting flowering days and each group contains 10 accessions. **C** The structure and haplotype analysis of the candidate genes with differential expression between the two groups. **D** Distribution of flowering time in six haplotypes of *AP1* (Glyma.02G100800.1). Different letters represent the significant levels at *P* < 0.05 based on one-way *ANOVA*

At chromosome 2, we found that there is a significant SNP peak (Chr02:9490318, *P* = 3.34 × 10^− 33^), and within the linkage disequilibrium block, there are 10 genes spanning from 9.4 to 9.5 Mb (Fig. 4A; Table S5). Based on differential gene expression analysis on 20 wild soybean accessions with contrasting flowering time (Table S2), we found that there are 1 gene significantly altered including *AP1* (Glyma.02G100800), which is annotated as Aspartic peptidase. In particular, the gene expression of *AP1* is 45% higher in late flowering subgroups, than that in early flowering subgroups (Fig. 4B). The suggestive threshold was used to determine the association at lower levels to identify the haplotype variation of candidate genes. In this regard, we found four SNPs above the suggestive threshold that significantly associated with the flowering time (Fig. 4C). Among these SNPs, three SNPs located on the exon of *AP1*, while one SNP located in intron, but there is no SNP significantly associated with flowering time on promoter region. Furthermore, 6 haplotypes were divided containing 41, 69, 27, 71, 40 and 46 accessions from haplotype (hap) 1 to 6, respectively (Fig. 4D). The flowering time in both hap 2 and hap 6 are significantly later than that in hap 4. In hap 6, 35% accessions were derived from AAT zone 1. In hap 4, 20% accessions were derived from AAT zone 1 (Fig. S6A).

**Fig. 5:**
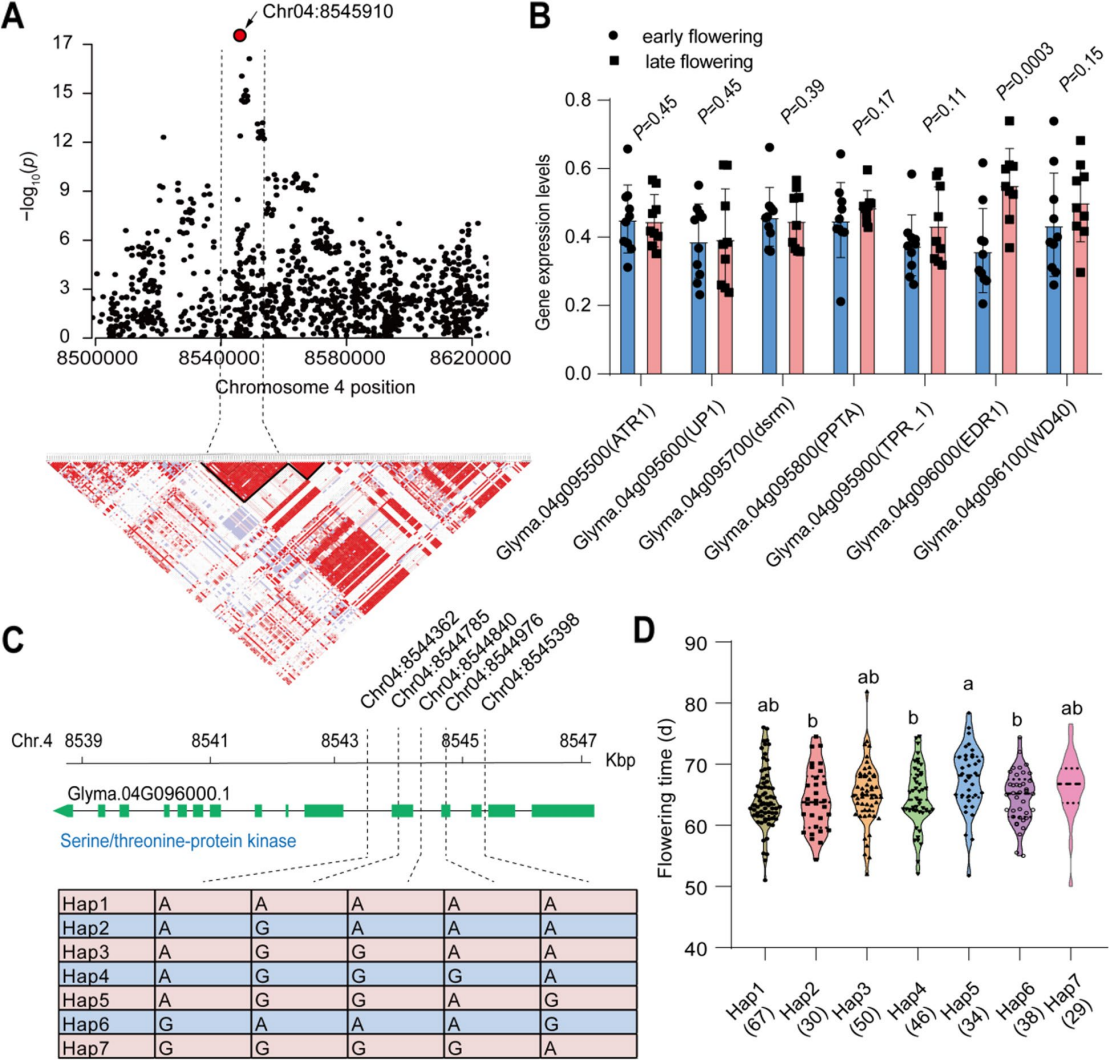
Haplotype analysis of aspartic peptidase at chromosome 4. **A** Zoom-out Manhattan plot of flowering days in average of 2 years at chromosome 4. The dotted lines represent the linkage disequibriurm (LD) (~ 23.09 kb) window surrounding the peaked SNP (Chr04:8545910). The red circle represents the lead SNP with -log_10_(*p*-value) = 33.59. **B** The relative gene expression levels of seven candidate genes within the LD window in 20 wild soybean accessions. The 20 accessions in two groups were characterized by their contrasting flowering days and each group contains 10 accessions. **C** The structure and haplotype analysis of the candidate genes with differential expression between the two groups. **D** Distribution of flowering time in six haplotypes of *EDR1* (Glyma.04G096000.1). Different letters represent the significant levels at *P* < 0.05 based on one-way *ANOVA*

At chromosome 4, we observed a significant SNP peak (Chr04:8545910, *P* = 3.35 × 10^− 33^) (Fig. 5A). Within the same linkage disequilibrium block, there are 7 genes located between 8.54 to 8.55 Mb (Fig. 5A; Table S5). Based on differential gene expression analysis on the 20 wild soybean accessions with contrasting flowering time (Table S2), we found that there is only one gene significantly altered, i.e., *EDR1* (Glyma.04 g096000), annotated as serine/threonine-protein kinase. The gene expression is 54% higher in late flowering subgroups than that in early flowering subgroups (Fig. 5B). Furthermore, we found that five SNPs that significantly associated with the flowering time (Fig. 5C). Among these SNPs, two SNPs located on the exon of *EDR1*, while three SNPs located in intron, but there is no SNP significantly associated with flowering time on promoter region. Moreover, 7 haplotypes were divided containing 67, 30, 50, 46, 34, 38 and 29 accessions from hap 1 to 7, respectively (Fig. 5D). The flowering time in hap 5 is significantly later than that in hap 2, hap 4 and hap 6. Within hap 5, approx. 25% accessions were derived from AAT zone 1, while in hap 2, 4 and 6, less than 20% accessions were derived from AAT zone 1 (Fig. S6B).

**Fig. 6:**
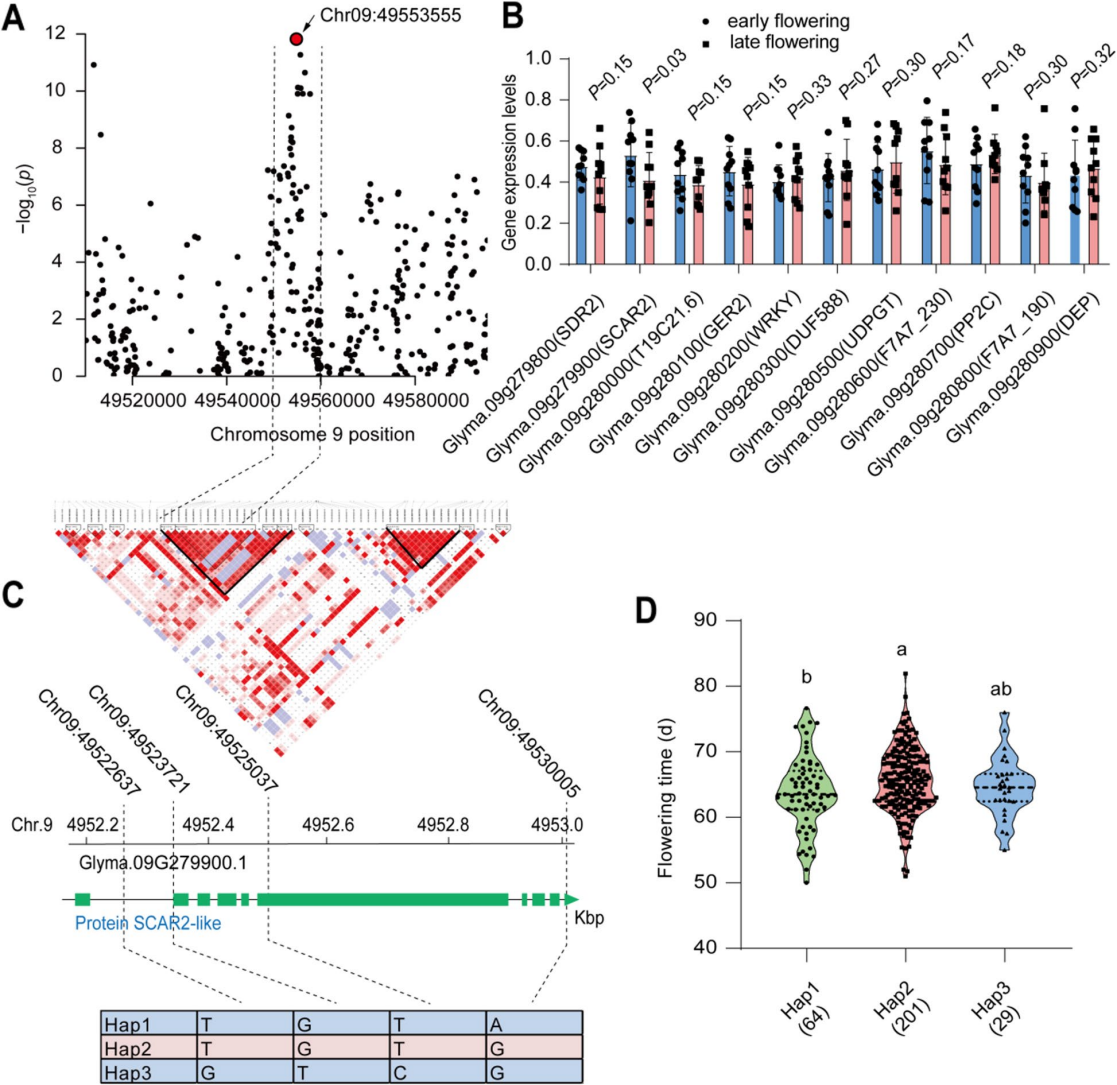
Haplotype analysis of aspartic peptidase at chromosome 9. **A** Zoom-out Manhattan plot of flowering days in average of 2 years at chromosome 9. The dotted lines represent the linkage disequibriurm (LD) (~ 37.72 kb) window surrounding the peaked SNP (Chr09:49553555). The red circle represents the lead SNP with -log_10_(*p*-value) = 32.15. **B** The relative gene expression levels of seven candidate genes within the LD window in 20 wild soybean accessions. The 20 accessions in two groups were characterized by their contrasting flowering days and each group contains 10 accessions. **C** The structure and haplotype analysis of the candidate genes with differential expression between the two groups. **D** Distribution of flowering time in six haplotypes of *SCAR2*(Glyma.09G279900.1). Different letters represent the significant levels at *P* < 0.05 based on one-way *ANOVA*

At chromosome 9, we observed a significant SNP peak (Chr09:49553555, *P* = 3.21 × 10^− 32^) (Fig. 6A). Within the same linkage disequilibrium block, there are 11 genes located between 49.5 to 49.6 Mb (Fig. 6B; Table S5). Based on differential gene expression analysis on the 20 wild soybean accessions with contrasting flowering time (Table S2), we found that there is also only one gene significantly altered, i.e., *SCAR2* (Glyma.09 g279900), annotated as SCAR2-like protein. The gene expression is 30% higher in early flowering subgroups, than that in late flowering subgroups (Fig. 6B). Furthermore, we found that four SNPs that significantly associated with the flowering time (Fig. 6C). Among these SNPs, two SNPs located on the exon of SCAR2, while two SNPs located in intron, but there is no SNP significantly associated with flowering time on promoter region. Furthermore, three haplotypes were divided containing 64, 201 and 29 accessions from hap 1 to 3, respectively (Fig. 6D). The flowering time in hap 2 is significantly later than that in hap 1. Within hap 2, 30% accessions were derived from AAT zone 1, while in hap 1, only 22% accessions were derived from AAT zone 1 (Fig. S6C).

## Discussion

Flowering time and AAT play crucial roles in the geographical adaptation of soybean. However, the molecular mechanism of flowering time incorporated with AAT is less reported. Flowering time trait is influenced by a large number of QTLs, but only a few genes controlling flowering time have been cloned and functionally identified [10, 24, 47]. Therefore, identifying additional genes involved in controlling flowering time will shed light on soybean adaptation and facilitate yield improvement. Breeding efforts of cultivated soybean are difficult to make further progress due to its genetic bottleneck effects. Compared with cultivated soybean, wild soybean has many excellent characters. In this study, we investigated the flowering time in 294 wild soybean accessions, and identified several novel gene/genetic loci using GWAS. This study provides a promising list of candidate genes that may be responsible for flowering time in wild soybean.

AAT is one of the most important environmental factors affecting flowering in plants. AAT plays an important role in agricultural research and impacts vegetation growth [46]. Due to the limitation of meteorological station distribution, most researchers investigated the flowering time irrespective of AAT effects. The relationship between AAT and the flowering time is poorly understood. Generally, the vegetative phase will stop transferring to reproductive growth (flowering prolonged) if the temperature is above the maximum base temperature or below the minimum base temperature [36]. This would definitely affect the final production. In current study, we observed a clearly negative effect of AAT on flowering time recorded in two-year experiments in the same location (Fig. 1C). As experiments conducted in this study in Harbin, Northeast of China. Harbin belongs to AT I with >2700C° of annual temperature [26]. The flowering time of accessions collected from AAT 4 and 5 were significantly shorter than that collected from AT 1 and 2, suggesting the environments of AT shapes the fitness of flowering time, as observed in other ecophysiology traits, such as stomatal features [15] and photosynthetic parameters [33].

Precise timing of flowering is critical to the environmental adaptation and productivity of crops [30]. In the photoperiodic pathway, FLOWERING LOCUS T (FT), a key photoperiod-regulated flowering integrator, encodes florigen which functions as a leaf-derived long-distance mobile signals and promotes floral transition [43]. In this study, we found three known QTLs related to flowering time at adjacent region of SNP with lower *P* values associated with flowering trait in wild soybean, such as GmFT2(Glyma.16 g150700), GmFT5(Glyma.16 g044100) and GmFT3(Glyma.16 g044200) (Table S4). These QTLs were previously reported to play coordinated roles in flowering promotion by up-regulating the expression of floral determination genes [24]. These results suggest the reliability of using this LRM model for flowering time in this study. Very interestingly, we found three common SNP peaks occurred in GWAS results of flowering traits performed in 2020, 2021 and their average, although the Pearson correlation coefficient of flowering traits between 2020 and 2021 was only 0.27. We further confirmed the results by analyzing haplotypic distribution of three candidate genes using the flowering time data recorded in 2020 and 2021 (Fig. S7). These evidences suggest that the association between differentially misclassified phenotypes and SNPs may strongly depend on the gene specificity [37].

**Fig. 7:**
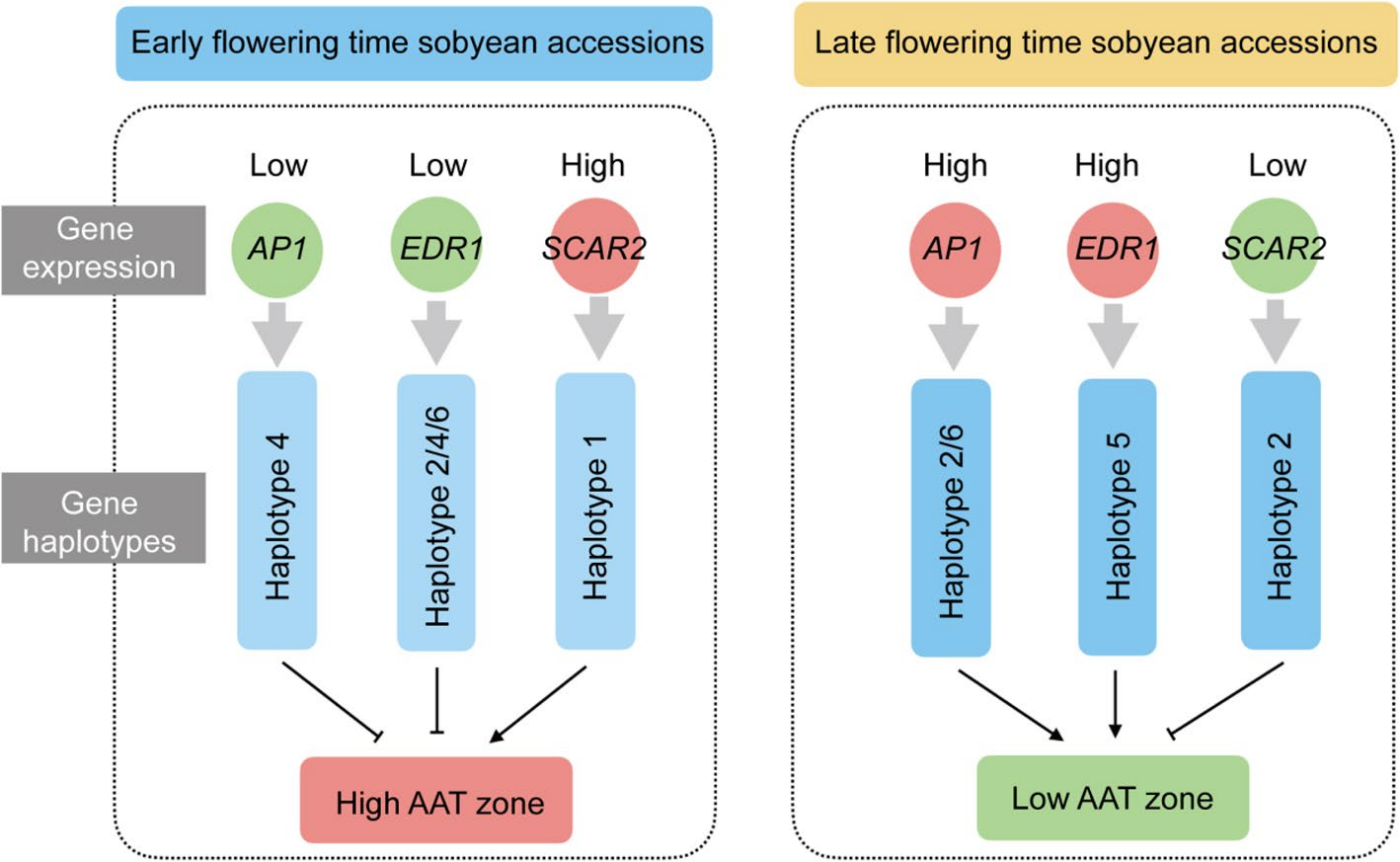
Working model of the genetic mechanism of three candidate genes regulating flowering time in response to AAT in wild soybean. The circle in red and green were used to represent the high and low gene expression in soybean accessions, respectively. High AAT zone stands for AAT zone with high active accumulated temperature such as AAT 5, while low AAT zone are AAT zone with low active accumulated temperature such as AAT 1. The haplotypes for each gene associated with early flowering time and late flowering time were displayed by light and dark blue colors, respectively

In addition, we found that most accessions possessing the haplotypes of two candidate genes (*AP1* and *EDR1*) with late flowering phenotypes were derived from AT 1, rather than AT 4 and 5, suggesting the two genes are positive regulator for flowering time (Fig. 7), and the genetic variation of the two genes could experience natural selection pressure. This is also observed in other flowering time regulatory genes, such as the major flowering time gene *Hd1* during domestication in cereal crops [25]. A loss of photoperiod sensitivity occurred in *COL2* and *PHYA3* function caused by natural selection in common bean [16]. The *Tof5H2* allele was selected via natural selection in wild soybean [10].

Aspartic peptidase (*AP1*, Glyma.02G100800) is a group of proteolytic enzymes that exist widely in bacteria, fungi, animals and plants. There are fifty-one potential APs found initially in Arabidopsis (*Arabidopsis thaliana*) and divided into three categories: typical APs, nucellin-like APs and atypical APs [12]. Although APs have been found in plants, knowledge of their functions is still lacking. It was reported that misexpression of the *AP1* gene alters leaf morphology and causes a delayed flowering time [31]. Consistently, we found that the wild soybean accessions with early flowering show lower expression of *AP1* than the accessions with late flowering phenotype (Fig. 4B). Therefore, we proposed that the *AP1* identified in our study is very likely to be a positive regulator involved in flowering signal pathway.

Serine-threonine protein kinase is reported to regulate salt stress-responsive gene expression in rice [9], and osmotic stress in tobacco and *A. thaliana* [18, 45]. A serine-threonine protein kinase, *EDR1* gene (Glyma.04 g096000) identified in this study shows higher expression in late flowering characterized soybean accessions compared to that in early flowering accessions (Fig. 5B). Consistently, the expression of its orthologs in Arabidopsis (AT4G24480) was induced during the floral initiation process in the soybean shoot apical meristem (SAM) [44]. We hence proposed that *EDR1* gene is positively regulator in flowering time in wild soybean species. Further study is required to confirm the biological function in this case.

The ARP2/3 complex, a highly conserved nucleator of F-actin, and its activator, the SCAR complex, are essential for growth in plants and animals [11]. In Arabidopsis, the *SCAR* is reported to mediate light-induced root elongation through photoreceptors and proteasomes. In current study, we found that the expression level of *SCAR2* (Glyma.09 g279900) gene was significantly higher in early flowering subgroups than that in late flowering subgroups. The mutant of its orthologs in Arabidopsis occasionally exhibit severe cell expansion and adherence defects such that in some instances cells tear out of the epidermal tissue layer and bend away from the surface [41], suggesting the *SCAR2* might have multiple functions in wild soybean. Therefore, the *SCAR2* gene is probably a negative regulator for flowering time, and the negative correlation of AAT on flowering time is also related to the hap 2 of *SCAR2* gene.

## Conclusion

Wild soybean provides abundant genetic resources for cultivated soybean research. In this study, we recorded the flowering time in 2 years in 294 soybean accessions derived from 4 AAT zones in Northeast of China. GWAS results suggest 10 overlapped SNPs that significantly associated with different sets of flowering time in 2020 and 2021 as well as flowering time in average in 2020–2021. Collectively, we identified three differentially expressed genes in 20 soybean accessions based on qPCR. Most accessions in subgroup of late flowering haplotypes belong to AAT zones with high ranking, suggesting that AAT shapes the flowering time and fitness of wild soybean. This study provides deep understanding of molecular mechanism of wild soybean adaption to AAT, which could be helpful to guide the cultivated soybean breeding.

## Supplementary Information

**Additional file 1: Table S1.** Prier used in this study. **Table S2.** List of 392 Glycine soja accessions and acumulated temperature zones. **Table S3.** 92 SNPs that significantly associated with flowering time in average across the two-year experiment (2020 and 2021). **Table S4.** The genes conresponding to the genomic regions surrounding ~50Kb of 92 SNPs that signficantly associated with flowering time in average in 2020 and 2021. **Table S5.** 28 candidate genes related to flowering time recorded in 2020 and 2021, and flowering time in average between 2020 and 2021. **Table S6.** Gene Ontology analysis on the 339 genes mapped to Arabidopsis gene ID.

**Additional file 2: Fig. S1.** Distribution of SNP number in different chromosomes and count of MAF. A, SNP numbers. B, SNP frequency in different minor allele frequency. **Fig. S2.** Distributions of SNP linkage disequilibrium decay and heterozygosity. A, Linkage disequilibrium decay (*r*^2^) across different linkage disequilibrium windows in 294 wild soybean accessions. B, Histogram of heterozygosity for SNPs. **Fig. S3.** Boxplot representing the distribution of flowering time in five active accumulated temperature zones in Heilongjiang province, China. A, flowering time data recorded in 2020; B, flowering time data recorded in 2021. Active accumulated temperature zones have five types (I, II, III, IV, and V). **Fig. S4.** Histogram of flowering time recorded from two consequent years. A, 2020 phenotyping of flowering days. B, 2021 phenotyping of flowering days. **Fig. S5.** Correlation of flowering time between two-year experiments in 2020-2021. Pearson correlation coefficient (*r*) was used. **Fig. S6.** Haplotype distribution of candidate genes against active accumulated temperature (AAT) zones. A, Glyma.02g100800. B, Glyma.04g096000. C, Glyma.09g279900. **Fig. S7.** Haplotypic distribution of flowering time in 2020-2021 for three candidate genes. A-B, TAXi_N haplotypes analysis in 2020 and 2021. C-D, EDR1 haplotypes analysis in 2020 and 2021. E-F, SCAR2 haplotypes analysis in 2020 and 2021. Different letters represent the significant levels at *P* <0.05 based on one-way *ANOVA*.

## Data Availability

All data is available in the manuscript or the supplementary materials. The sequencing data of 294 accessions used in this study have been deposited into the NCBI database under accession number PRJNA743225 (https://www.ncbi.nlm.nih.gov/bioproject/PRJNA743225).
