# Supplementary material for: Genome-wide association study uncovers major genetic loci associated with flowering time in response to active accumulated temperature in wild soybean population
Source: BMC Genomics. 2022 Nov 11;23:749. doi: 10.1186/s12864-022-08970-2 (PMC9652842; doi:10.1186/s12864-022-08970-2)
Supplement: Supplementary file 2 — Additional file 2: Fig. S1. Distribution of SNP number in different chromosomes and count of MAF. A, SNP numbers. B, SNP frequency in different minor allele frequency. Fig. S2. Distributions of SNP linkage disequilibrium decay and heterozygosity. A, Linkage disequilibrium decay (r2) across different linkage disequilibrium windows in 294 wild soybean accessions. B, Histogram of heterozygosity for SNPs. Fig. S3. Boxplot representing the distribution of flowering time in five active accumulated temperature zones in Heilongjiang province, China. A, flowering time data recorded in 2020; B, flowering time data recorded in 2021. Active accumulated temperature zones have five types (I, II, III, IV, and V). Fig. S4. Histogram of flowering time recorded from two consequent years. A, 2020 phenotyping of flowering days. B, 2021 phenotyping of flowering days. Fig. S5. Correlation of flowering time between two-year experiments in 2020-2021. Pearson correlation coefficient (r) was used. Fig. S6. Haplotype distribution of candidate genes against active accumulated temperature (AAT) zones. A, Glyma.02g100800. B, Glyma.04g096000. C, Glyma.09g279900. Fig. S7. Haplotypic distribution of flowering time in 2020-2021 for three candidate genes. A-B, TAXi_N haplotypes analysis in 2020 and 2021. C-D, EDR1 haplotypes analysis in 2020 and 2021. E-F, SCAR2 haplotypes analysis in 2020 and 2021. Different letters represent the significant levels at P <0.05 based on one-way ANOVA. [file 12864_2022_8970_MOESM2_ESM.docx]

**Supplementary Figures**


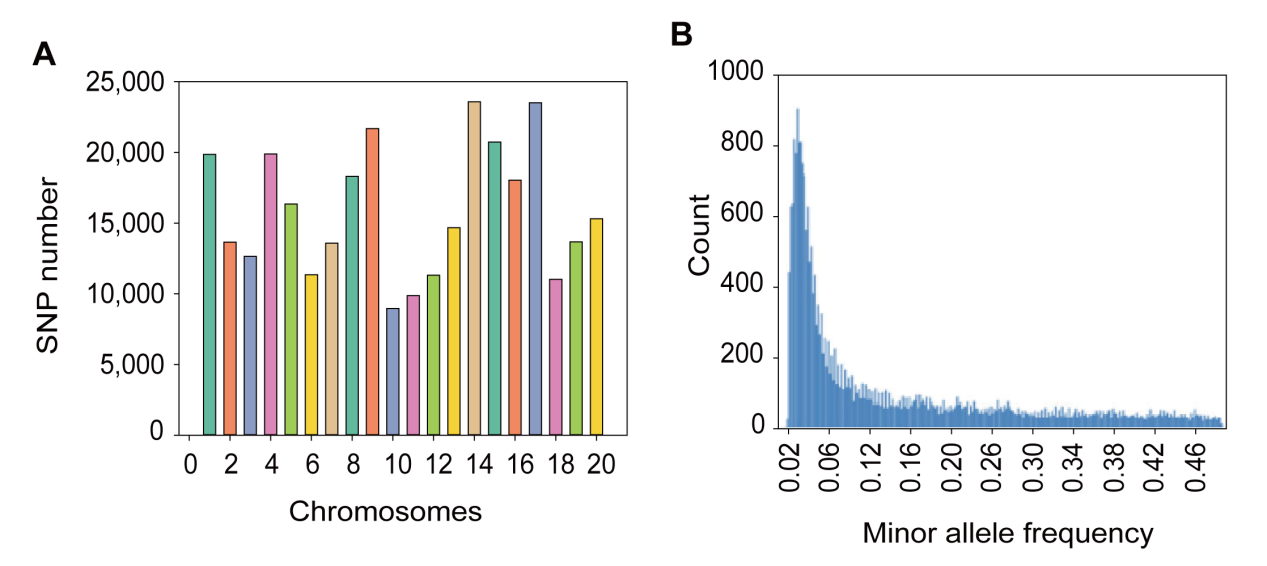


**Fig. S1** Distribution of SNP number in different chromosomes and count of MAF. **A**, SNP numbers. **B**, SNP frequency in different minor allele frequency.


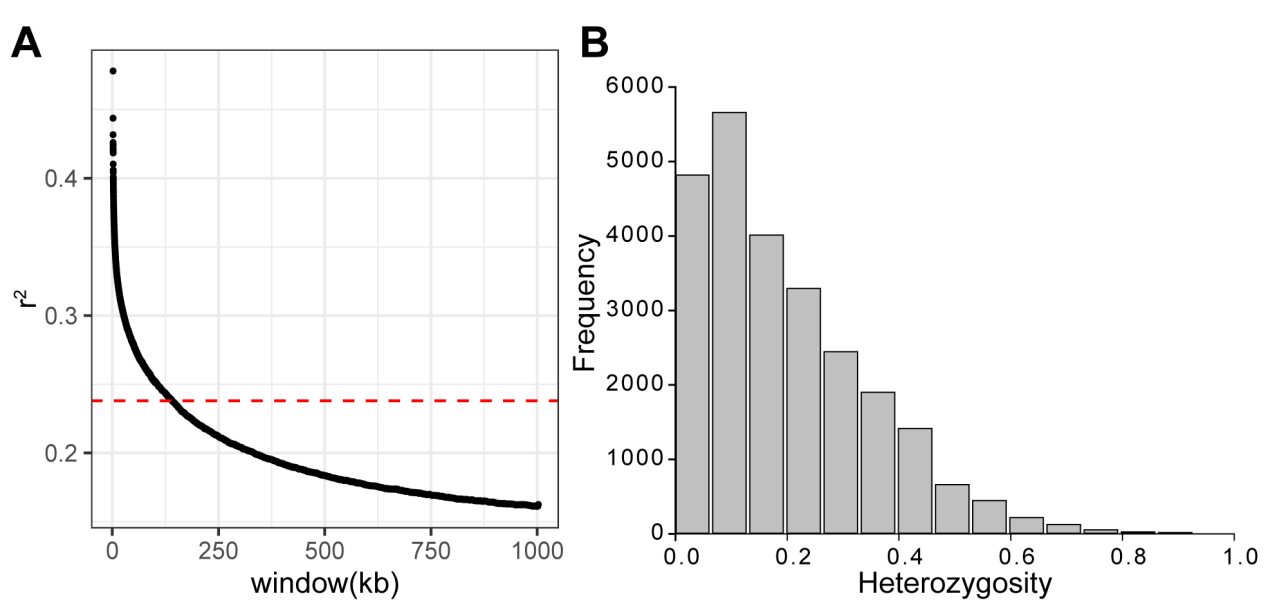


**Fig. S2** Distributions of SNP linkage disequilibrium decay and heterozygosity. **A**, Linkage disequilibrium decay (*r*^2^) across different linkage disequilibrium windows in 294 wild soybean accessions. **B**, Histogram of heterozygosity for SNPs.


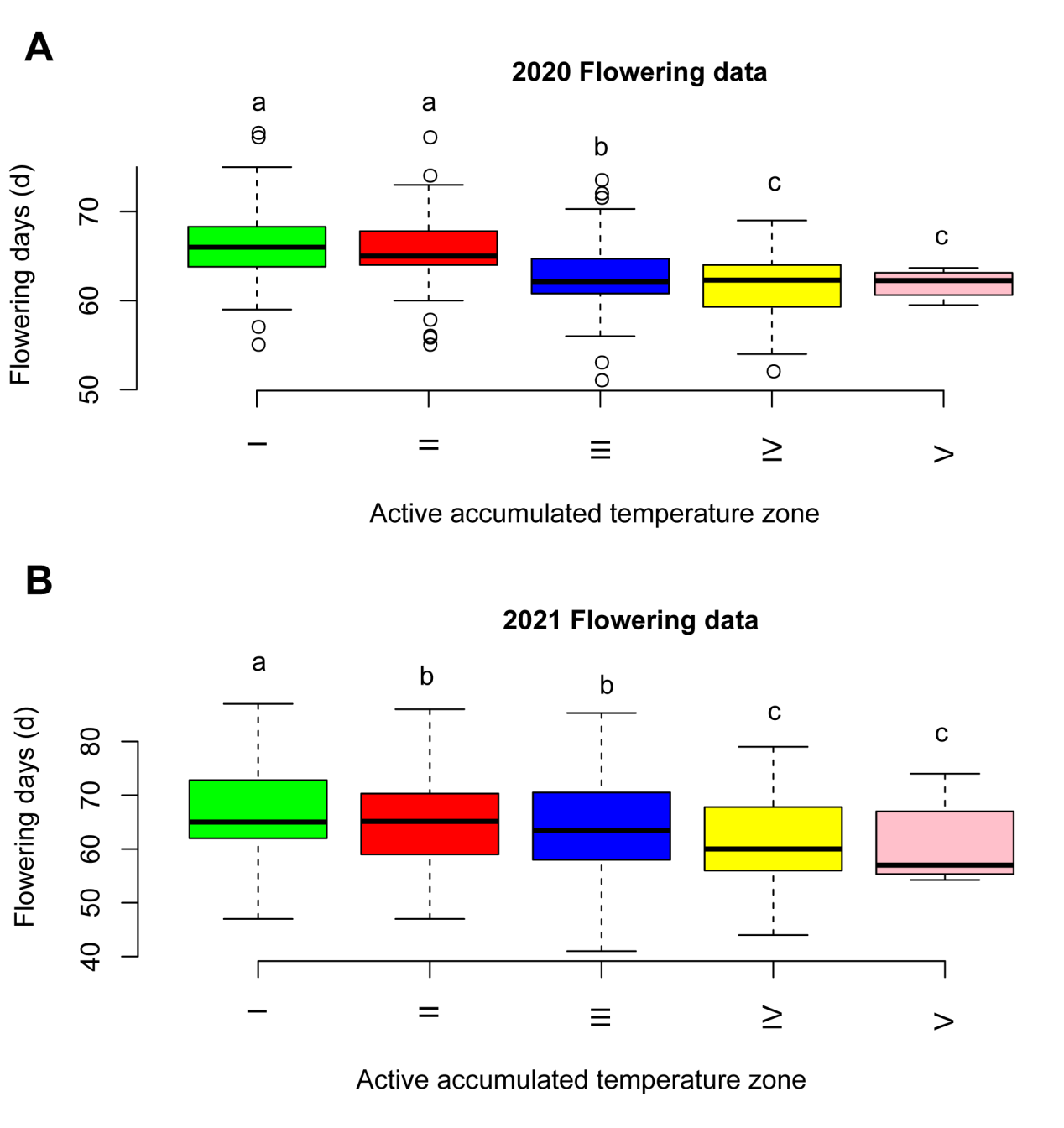


**Fig. S3** Boxplot representing the distribution of flowering time in five active accumulated temperature zones in Heilongjiang province, China. **A**, flowering time data recorded in 2020; **B**, flowering time data recorded in 2021. Active accumulated temperature zones have five types (I, II, III, IV, and V).


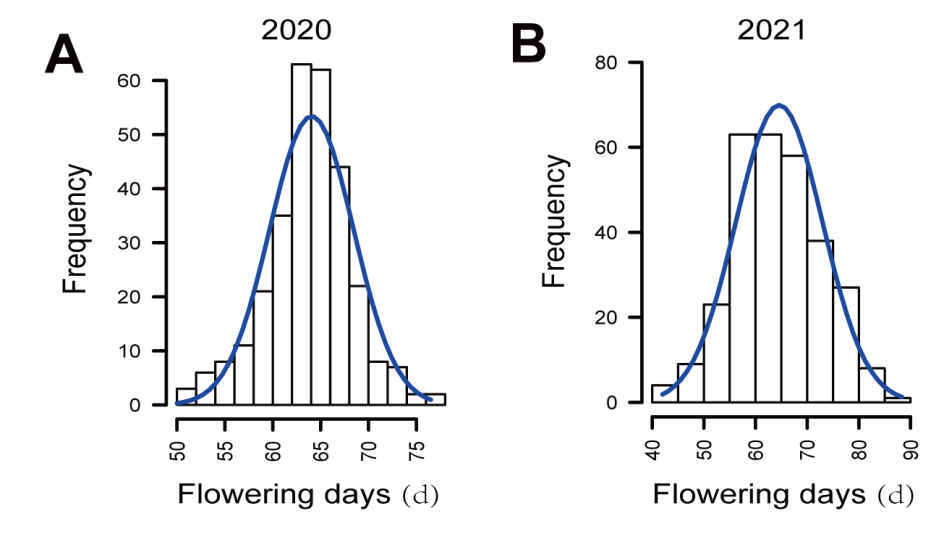


**Fig. S4** Histogram of flowering time recorded from two consequent years. **A**, 2020 phenotyping of flowering days. **B**, 2021 phenotyping of flowering days.


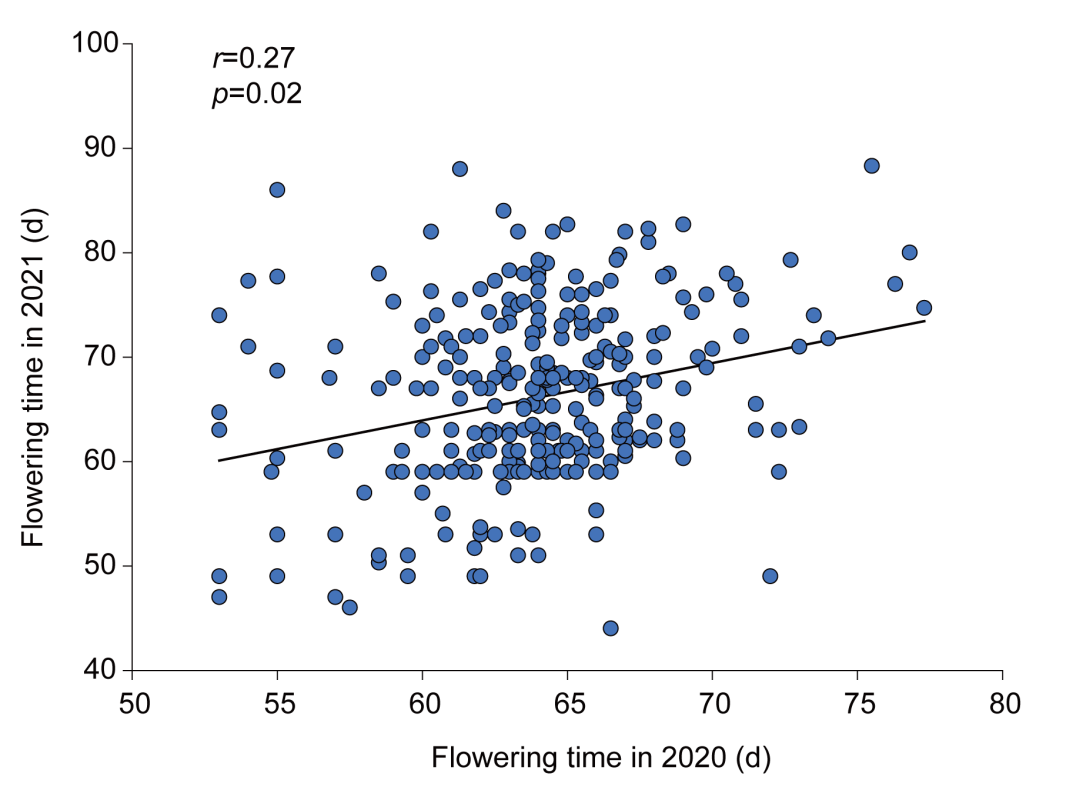


**Fig. S5** Correlation of flowering time between two-year experiments in 2020-2021. Pearson correlation coefficient (*r*) was used.

**
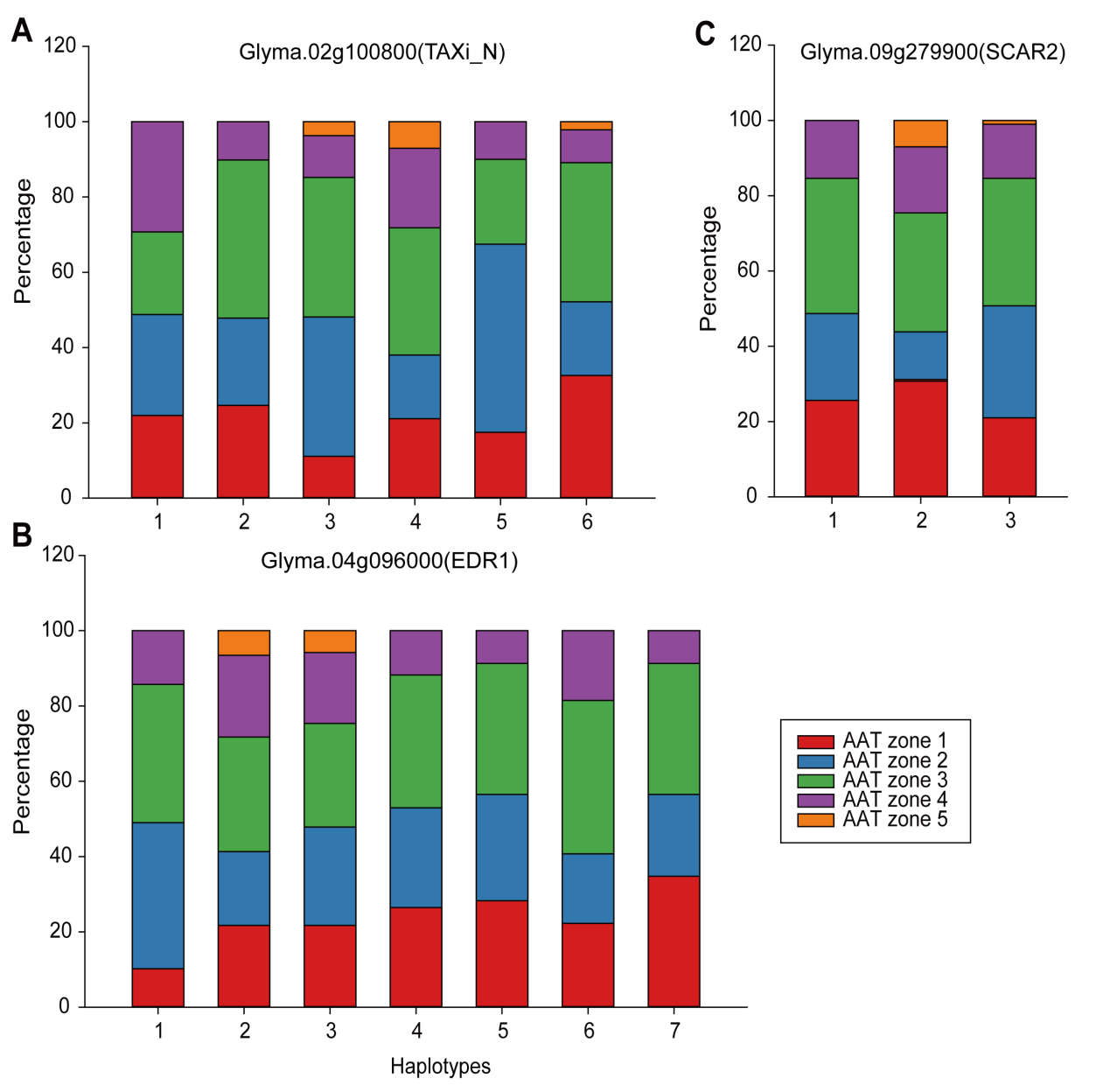
**

**Fig. S6.** Haplotype distribution of candidate genes against active accumulated temperature (AAT) zones. **A**, Glyma.02g100800. **B**, Glyma.04g096000. **C**, Glyma.09g279900.


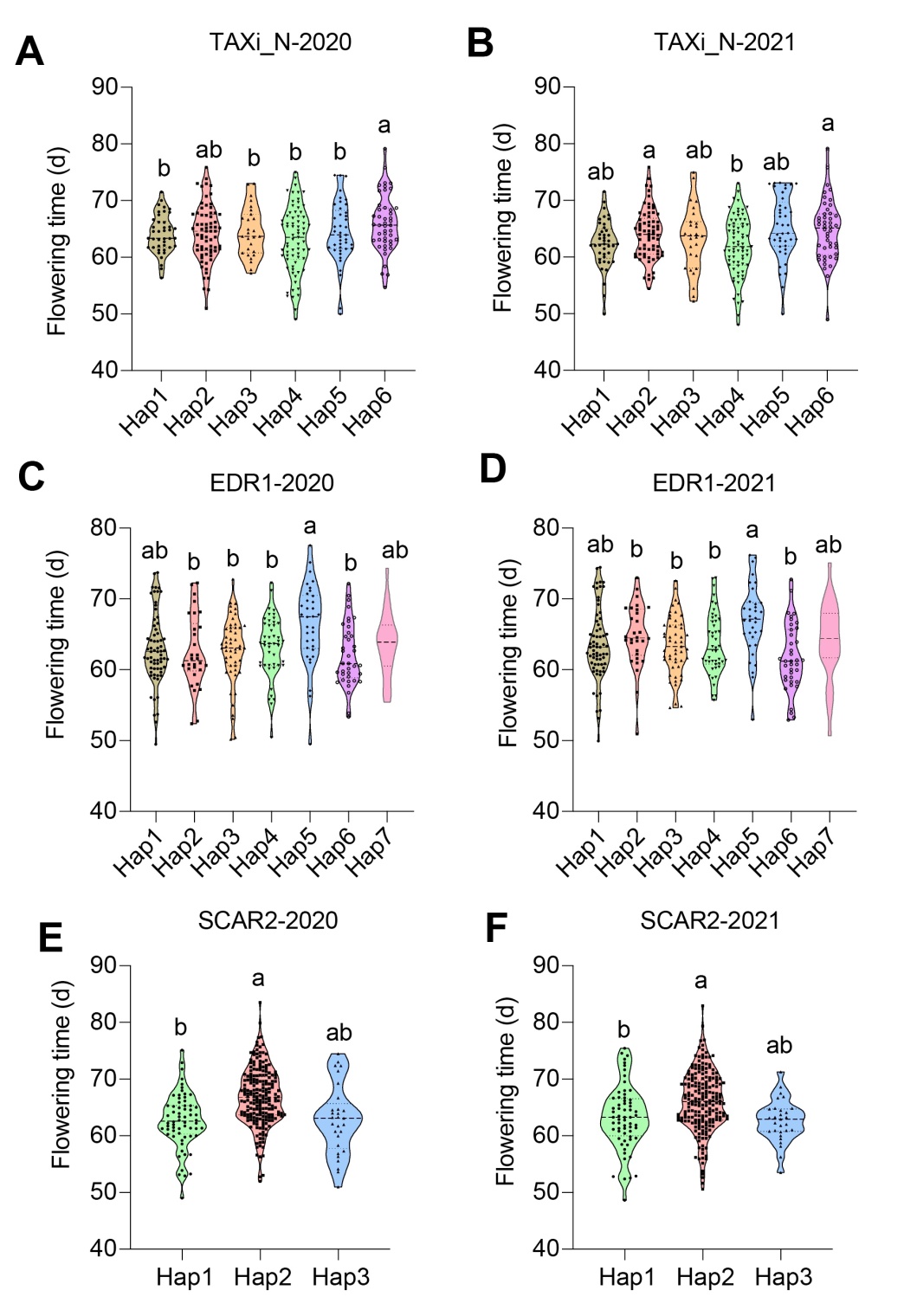


**Fig. S7.** Haplotypic distribution of flowering time in 2020-2021 for three candidate genes. **A-B,** TAXi_N haplotypes analysis in 2020 and 2021. **C-D,** EDR1 haplotypes analysis in 2020 and 2021. **E-F,** SCAR2 haplotypes analysis in 2020 and 2021. Different letters represent the significant levels at *P* <0.05 based on one-way *ANOVA*.
